# Endothelial cells, neutrophils and platelets: getting to the bottom of an inflammatory triangle

**DOI:** 10.1098/rsob.200161

**Published:** 2020-10-14

**Authors:** Tima Dehghani, Alyssa Panitch

**Affiliations:** Department of Biomedical Engineering, University of California, Davis, 451 Health Sciences Drive, GBSF 2303, Davis, CA, USA

**Keywords:** neutrophil, platelet, endothelial dysfunction, inflammation, fibrosis, glycocalyx

## Abstract

Severe fibrotic and thrombotic events permeate the healthcare system, causing suffering for millions of patients with inflammatory disorders. As late-state consequences of chronic inflammation, fibrosis and thrombosis are the culmination of pathological interactions of activated endothelium, neutrophils and platelets after vessel injury. Coupling of these three cell types ensures a pro-coagulant, cytokine-rich environment that promotes the capture, activation and proliferation of circulating immune cells and recruitment of key pro-fibrotic cell types such as myofibroblasts. As the first responders to sterile inflammatory injury, it is important to understand how endothelial cells, neutrophils and platelets help create this environment. There has been a growing interest in this intersection over the past decade that has helped shape the development of therapeutics to target these processes. Here, we review recent insights into how neutrophils, platelets and endothelial cells guide the development of pathological vessel repair that can also result in underlying tissue fibrosis. We further discuss recent efforts that have been made to translate this knowledge into therapeutics and provide perspective as to how a compound or combination therapeutics may be most efficacious when tackling fibrosis and thrombosis that is brought upon by chronic inflammation.

## 1. Introduction

The peripheral vasculature is a complex continuum made up in part by a collagenous tunica externa, an elastic and smooth muscle-lined tunica media and an endothelial monolayer making up the tunica intima, or inner vessel lining. This structure differs slightly in the capillaries, which are composed of a tunica intima and basement membrane. Vascular integrity relies on coordinated homeostasis of these dynamic components. As the inner lining of the blood vessel, the endothelium provides an interface between circulating blood components and the adjacent tissues and plays a particularly pivotal role in tissue damage and fibrosis. In response to vascular damage, metabolic disorders or inflammation, endothelial cells prompt the recruitment of key inflammatory cells: platelets and neutrophils. Changes to endothelial morphology, chemokine production and surface proteins foster a pro-inflammatory environment that mediates the mobilization of circulating immune cells such as polymorphonuclear neutrophils and platelets. In many circumstances, interactions between recruited neutrophils, platelets and the endothelium lead to the resolution of vessel damage; indeed, inflammatory events are vital for proper wound healing [1]. However, if inflammatory stimuli are persistent these interactions can tip the balance toward fibrotic healing.

The fibrotic response is the culmination of many chronic inflammatory diseases. Fibrosis is characterized as the accumulation of excess extracellular matrix (ECM) proteins, such as collagen and fibronectin. Although ECM deposition is a vital and largely self-restorative part of wound healing, repetitive or severe damage can cause pathological dysregulation of this process leading to fibrosis. Inflammation mediated by the innate immune response is a critical trigger of pathological fibrosis [2]. In response to vascular damage or systemic and local inflammation, endothelium transitions from a quiescent state to a state called endothelial dysfunction, which in its severe form causes the exposure of underlying ECM such as collagen [3]. Soluble pro-inflammatory factors such as reactive oxygen species (ROS) and matrix metalloproteinases (MMPs) are released by the endothelium [4,5], recruiting circulating neutrophils to the scene. Neutrophils and circulating platelets tether to upregulated cell adhesion molecules that are expressed on inflamed endothelium, such as E-selectin, P-selectin and von Willebrand Factor (vWF). This tethering initiates an inflammatory cascade that, if persistent, culminates as thrombotic vessel occlusion or fibrotic myofibroblast proliferation and ECM deposition. Left unchecked, excessive ECM deposition can result in impaired organ function and, in some cases, end-stage organ failure and death.

**Figure 1.**
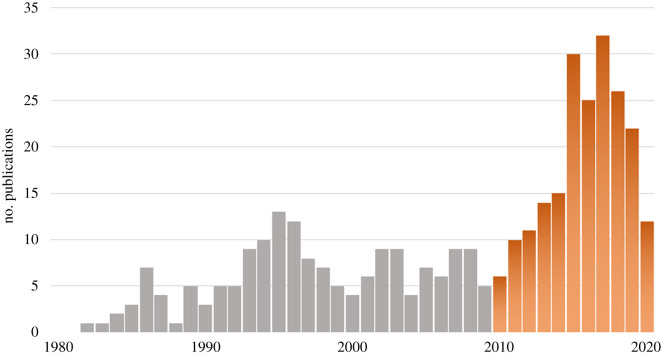
PubMed search results since 1980. A PubMed literature search for the years 1980–2020 was conducted for the following key terms: neutrophil, platelet, endothelial and thrombosis or fibrosis. Results were further narrowed down to the years 2010–2020, during which the number of publications per year steadily increased. Publications were screened for titles relevant to topics discussed in this review.

Given the influential roles endothelial cells, neutrophils and platelets play in aberrant inflammation, thrombosis and fibrosis, many studies have focused on elucidating mechanisms behind pro-fibrotic and prothrombotic events in order to develop targeted therapeutics that interfere with these processes. These cell types have been of growing interest over the past decade, as is evidenced by the steady increase in the relevant literature (figure 1). In this review, we detail how disruptions to the endothelium and its protective ECM layer, the glycocalyx, contribute to endothelial cell dysfunction, neutrophil and platelet dysregulation, thrombosis, and fibrosis. We discuss the promise and limitations of therapeutic strategies for limiting neutrophil and platelet-mediated fibrosis.

### 1.1. Endothelial dysfunction within the scope of inflammation

The blood vessels are a conduit for immune cell trafficking in response to tissue damage. Amid differences in vessel composition, elasticity, size and stiffness, all blood vessels are lined by an inner layer of endothelium. The vascular endothelium is made up of a heterogeneous continuum of individual endothelial cells that reside on a bed of collagen-rich ECM [6]. As its most basic function, the endothelium acts as a physical barrier between circulating fluids and the surrounding tissues. For many years, this was believed to be the sole purpose of the endothelium. It is now well accepted that the endothelium is a complex, bioactive cell layer that regulates immune cell function and tissue access, vasoreactivity, and the extravasation of macromolecules, solutes, hormones and fluids [7]. Endothelial cell health plays a precarious role in chronic inflammatory and thrombogenic diseases, including diabetes [8], atherosclerosis [4,9], acute respiratory distress syndrome [10], cystic fibrosis [11] and liver cirrhosis [12]. Damage to the endothelium initiates and sustains inflammation and thrombogenesis [7], with endothelial cells themselves providing a platform to fast-track thrombosis during dysregulated haemostasis, and fibrosis during pathological wound repair.

The endothelium is lined by a thin, glycosaminoglycan-rich barrier called the endothelial glycocalyx. The glycocalyx is a dynamic participant in endothelial cell barrier function and immune regulation [13]. Structurally, the glycocalyx ranges from nanometres to several micrometres thick, depending on the vessel type, location within a vessel, conditions and the imaging technique used [14,15]. Even within one vessel, the glycocalyx is a heterogeneous structure [16]. The glycocalyx is comprised a glycoprotein or proteoglycan core protein anchored to underlying actin cytoskeletal filaments at the endothelial surface [13,17]. While the luminal portion of a glycoprotein is decorated with small sugar residues, the proteoglycan core, primarily from the syndecan and glypican families [16,18], extends into the vessel lumen and is decorated with glycosaminoglycans (GAGs) such as heparan sulfate, chondroitin sulfate, hyaluronan and dermatan sulfate. GAG components, particularly heparan sulfate and dermatan sulfate, possess anticoagulant and anti-thrombotic qualities [19,20] that have been used as therapeutics [21–24]. Though heparan sulfate makes the greatest contribution to glycocalyx thickness, heterogeneity within the glycocalyx suggests that each GAG contributes to glycocalyx function and vessel permeability [25].

Under healthy conditions, the glycocalyx exists in dynamic equilibrium with circulating blood, changing its composition and thickness in response to haemodynamic forces and cues from soluble factors [26] in order to maintain equilibrium between hydrostatic and oncotic forces in the vessel [27]. Changes to the rheological environment are detected by the glycocalyx [28] and transduced to the underlying endothelium via actin anchoring. The actin cytoskeleton and the glycocalyx exist in a dynamic equilibrium with one another, each being modified in response to changes in the other to control endothelial barrier properties [29,30]. Indeed, mechanical stress has been shown to alter GAG and proteoglycan synthesis and remodelling in vascular endothelial cells [31,32], lending insight into how pathological stretch and disturbed flow can influence endothelial cell behaviour and function.

After physical, local or systemic inflammatory insult, damaged endothelium exhibit signs of endothelial dysfunction. Endothelial dysfunction is a complex phenomenon involving heightened ROS production, altered nitric oxide (NO) production and disruptions to vascular tone; production of MMPs and pro-inflammatory cytokines; and upregulation of cell adhesion molecules such as E-selectin, P-selectin and intracellular adhesion molecule-1 (ICAM-1) [4,5,33,34]. MMPs and ROS facilitate glycocalyx degradation and shedding, causing impaired mechanosensing and altering cell behaviour [22,28,35] to enable immune cell capture and trafficking [26,36–39]. Compromised mechanosensing can in turn perpetuate reductions in shear-sensitive NO secretion and augment vascular permeability. Paired with the release of sequestered chemokines and the exposure of immune cell adhesion molecules along the endothelial surface, glycocalyx degeneration facilitates inflammation, thrombosis and eventual fibrosis by promoting immune cell migration into the underlying tissue [40,41].

Dysfunctional endothelium plays an interesting role in perpetual inflammation. While in a dysfunctional state, endothelial cell senescence is accelerated and migration is hindered [31,42]. In damaged vessels, the inability to replenish the endothelial layer with healthy cells results in a prolonged inflammatory attack. Even when re-endothelialization can occur, newly formed glycocalyx is fragile and less responsive to shear [26], and readily concedes to the inflammatory state*.* Further exacerbating the dysfunctional state, circulating platelets bind to collagen, which normally resides below the surface of the endothelium [6,43], but is exposed between contracting cells [44] or is revealed after vessel denudation [45]. In some cases, adherent platelets induce a pro-fibrotic cascade involving platelet-mediated leucocyte recruitment, tissue factor secretion and fibrin(ogen) deposition [46,47].

## 2. Drivers of platelet–neutrophil aggregation, endothelial dysfunction and fibrosis

Inflammatory stimulus and vessel choice influence the relative contributions of platelets, neutrophils and endothelial cells to inflammation, thrombosis and fibrosis [48] (table 1). Here, we discuss recent insights into how these three cell types collaborate to exacerbate inflammation, thrombosis and fibrosis.

**Table 1.** Endothelial injury methods and outcomes. (HUVEC, human umbilical vein endothelial cells; PSGL-1, P-selectin glycoprotein ligand-1; Par4, protease-activated receptor 4; TNF-α, tumour necrosis factor alpha; IFN-γ, interferon gamma; CCL2, C-C motif chemokine ligand 2; IL-1β, interleukin-1 beta; ApoE, apolipoprotein E; TF, tissue factor.)

vessel or cell type	injury method	key observations	references
cremaster arteriole	laser activation	laser activated endothelial cells trigger thrombus formation; neutrophil slow rolling on thrombus mediated by P-selectin-PSGL-1	[49]
cremaster arteriole and HUVEC	laser activation	endothelial activation precedes platelet accumulation; normal fibrin formation observed in Par4^-/-^ mice	[50]
cremaster arteriole	laser activation	prothrombinase found on activated endothelial cells	[51]
cremaster arteriole	laser activation	neutrophils contain and express tissue factor at the site of laser injury; neutrophils accumulated before platelets	[52]
venule	TNF-α	TNF-α activated endothelial cells recruit neutrophils; platelets bind adherent neutrophils rather than endothelium	[49]
artery and HUVEC	TNF-α and IFN-γ	fractalkine causes degranulation, activation, and expression of platelet P-selectin on adherent platelets, mediating neutrophil recruitment	[53]
HUVEC	TNF-α	endothelial TF drives fibrin deposition and coagulation; upregulated ICAM can be targeted for delivering recombinant thrombomodulin to inflamed cells	[47]
cremaster arteriole	CCL2, TNF-α, IL-1*β*, or IFN-γ	platelets guide neutrophils to extravasation points via P-selectin-PSGL-1 and CD40/CD40 L	[54]
artery and HUVEC	ApoE^-/-^ mice	increased endothelial stiffness causes enhanced leucocyte transendothelial migration	[55]
artery	ApoE^-/-^ mice	reduced glycocalyx thickness and increased platelet adhesion occur at bifurcation point	[56]
artery	ApoE^-/-^ mice	endothelial dysfunction and glycocalyx impairment coincide with endothelial-dependent vasodilation, permeability, and increases in atherosclerotic biomarkers	[4]

### 2.1. Endothelial cells

As the lining of the conduits of blood components, metabolites and immune cells, the vascular endothelium is often on the front line in an inflammatory insult. A major source of endothelial inflammation is the overproduction of ROS, whose aetiology can range from diet, to hormone dysregulation, to cellular by-products such as those produced during macrophage digestion of apoptotic debris and foreign substances [34]. Excess ROS inactivates NO production—a known mediator of vascular tone. Reduced NO enhances vessel stiffness and contractility, both of which contribute to endothelial dysfunction [4]. Paradoxically, NO overproduction can likewise induce cellular inflammation and apoptosis [57], highlighting the importance of maintaining tight regulation of homeostasis.

Endothelial cytoskeletal structure and stiffness play a critical role in leucocyte recruitment and fibrosis. Tumour necrosis factor alpha (TNF-α) stimulated endothelial cells present a cortical stiffness gradient to slow-rolling neutrophils, guiding them to transmigration sites [55]. This effect is augmented on stiff matrices [55,58], consistent with neutrophil recruitment and extravasation in atherosclerotic vessels [9]. Pro-fibrotic cellular pathways such as the autotaxin/lysophosphatidic acid axis likewise stimulate endothelial actin rearrangement and cell contractility, causing a vascular leak and aiding in the migration of fibroblasts in the underlying tissue [59]. Structural alterations of the cytoskeleton affect glycocalyx distribution and function [29], further exacerbating endothelial dysfunction and vascular damage.

Dysfunctional endothelial cells are known initiators of fibrosis. In response to noxious stimuli such as excess ROS, lipopolysaccharide (LPS), bleomycin or physical damage, dysfunctional endothelial cells secrete a milieu of pro-inflammatory cytokines including TNF-α, IL-1β and IL-6 [2]. TNF-α initiates a signalling cascade that can lead to endothelial apoptosis or necrosis [60]. Surviving endothelial cells begin to shed their glycocalyx, unmasking upregulated selectins and integrin-binding ligands embedded within the cell membrane [61]. In the venules, selectins act as the initial point of neutrophil capture [62], which can then act as secondary capture sites for circulating platelets [49]. Bleomycin-induced pulmonary fibrosis exemplifies the potential pathological response once the endothelium becomes inflamed. In one representative study, bleomycin-induced pulmonary endothelial inflammation resulted in a peak in endothelial inflammation at 7 days post-bleomycin instillation; and enhanced vWF, plasminogen activator inhibitor-1, MMP-12 and NO that led to increased collagen deposition, and pulmonary fibrosis peaking at day 21, clearly delineating the link between endothelial dysfunction and fibrosis [57].

Similarly, studies using alternative stimuli have evidenced endothelial cells as sources of tissue factor (TF) [47,50,63], a protein that is primarily secreted by activated monocytes to initiate platelet deposition and thrombin formation [64] and thereby actuate thrombogenesis and tissue fibrosis [65,66]. Endothelial-derived TF has been reported in response to TNF-α [47,63] and laser activation [50,51], a technique which can elicit endothelial activation without vessel denudation that has been used as a model of vascular thrombosis and atherosclerosis [67]. Endothelial TF has been shown to cause fibrin deposition, upregulation of ICAM-1 and vascular cell adhesion molecule-1, and increased platelet binding. Atkinson *et al*. demonstrated that endothelial activation, calcium mobilization and granule secretion precede platelet accumulation in cremaster arterioles [50]. Notably, fibrin production persisted on activated human umbilical vein endothelial cells (HUVECs) upon treatment with platelet depleted plasma, further validating endothelial cells as a source of TF. Ivanciu and colleagues later observed enhanced prothrombinase activity on similarly activated endothelium, reinforcing the idea that activated endothelium forms a pro-coagulant surface that supports thrombus formation after injury [51].

#### 2.1.1. Link to the inflammatory triangle

Despite similar methods of endothelial activation, there is a lack of consensus regarding the main source of TF produced in response to vessel damage. Contrary to the results described above, leucocytes have been demonstrated as the first responders to endothelial activation. Adherent leucocytes express TF and create a platform for platelet binding [52]. In a separate study, neutrophil rolling and adhesion was observed only after platelet thrombi had formed, with rolling mediated by platelet P-selectin and neutrophil P-selectin glycoprotein ligand-1 (PSGL-1) [49], suggesting multiple factors are probably at play, orchestrating the timing and sequence of events.

While it is clear all three cell types work in coordination to address vessel damage, more studies are needed to elucidate mechanisms behind thrombo-inflammation in models of cytokine, bacterial and physical damage. Understanding these mechanisms may allow for the development of more targeted therapeutics to combat vascular inflammation and fibrosis, for example targeting endothelial—but not leucocyte or platelet-derived TF, or selectively upregulated cell adhesion molecules. Further investigation into these mechanisms from the perspective of neutrophils and platelets follows.

### 2.2. Neutrophils

Neutrophils play a paradoxical role in inflammation. The neutrophil recruitment cascade is a conserved process that is integral to the resolution of inflammation, infection and wound healing [68]. However, the pathological activation of neutrophils perpetuates acute and chronic inflammatory diseases. Pathological neutrophilia in response to cytokine storm is observed in rheumatic diseases, as well as infectious diseases such as coronavirus pneumonia seen in severe acute respiratory syndrome (SARS), Middle East respiratory syndrome (MERS) and coronavirus disease 2019 (COVID-19) [69,70], leading to irreversible tissue fibrosis or necrosis.

We have known for decades that neutrophil engagement exacerbates endothelial activation [71] and collagen synthesis [72]. Neutrophils are recruited to the endothelial surface in response to endothelial cues after cellular activation. As previously described, endothelial cells release a milieu of pro-inflammatory stimuli upon damage such as ROS, TNF-α, IL-1β and MMPs that degrade the endothelial glycocalyx and recruit circulating neutrophils to the damaged area. Tethered neutrophils roll along the endothelial surface, stabilized by shear-strengthening, transient catch bonds between endothelial E/P-selectin, CD44 and neutrophil PSGL-1 [61,73]. E/P-selectin engagement directs neutrophil signalling by stimulating an intracellular neutrophil calcium burst [74,75] and augments chemokine signalling to activate neutrophil *β*2 integrins, leading to cell arrest, migration into the underlying tissue, and further neutrophil recruitment.

With the exception of the pulmonary system, in which cell recruitment occurs at the capillaries [76,77], veins and post-capillary venules act as the primary capture sites for neutrophils during inflammatory damage, though arterial recruitment has been observed after physical vessel damage by laser activation [49–52,78]. This unequal involvement is perhaps owing to differences in junctional proteins that result in increased intrinsic leakiness within the venules, selectin and ICAM-1 expression, shear stress and flow mechanics, and gene expression [7,79]. In the venules, TNF-α and LPS activation quickly result in neutrophil adhesion to the vessel wall [48,49,80,81]. Adherent and activated neutrophils secrete endothelial barrier disrupting molecules such as ROS and release of neutrophil degranulation products including myeloperoxidase, elastase and metalloproteases [82], facilitating migration through the endothelium.

Further damage can be caused by neutrophil extracellular trap (NET) production. Coordinated PSGL-1 [83] and CXCR2 [73] signalling leads to enhanced neutrophil adhesion, NET formation and flow restriction. While their primary function is to trap pathogens, NETs have been shown to enhance inflammation and endothelial permeability and have been implicated in several inflammatory diseases [82]. Further, NETs are known to trap platelets, red blood cells and fibrin, fostering the growth of pathological thrombi. NETs are drivers of vascular damage, venous thrombosis [73,84] and virus-induced organ damage and mortality, as is seen in severe COVID-19 [69,85].

#### 2.2.1. Link to the inflammatory triangle

Neutrophil-mediated fibrin deposition and thrombosis can occur even in the absence of exogenous inflammatory stimuli or direct endothelial cell damage, such as in a murine stenosis model of deep vein thrombosis (DVT). In this model, the endothelium remains intact and the underlying collagen-rich ECM unexposed, but altered blood flow by partial vessel occlusion is enough to cause cooperative signalling by neutrophils at the endothelial surface that initiates fibrin formation and propagates venous thrombosis [73,84]. Once bound to the endothelium, neutrophils physically alter the rheological environment within the vasculature by creating an altered flow pattern near the vessel wall [48]. Drag forces grab circulating platelets and bring them towards the surface of the neutrophil, allowing molecular interactions between platelet and neutrophil. Interestingly, NETs have also been implicated in DVT and probably exacerbate neutrophil-mediated thrombosis [86].

This relationship between rheological haemostasis and thrombosis aligns with atherosusceptible vascular regions such as vessel branches, where blood flow patterns are disturbed [87]. However, rather than disturbed flow causing endothelial activation, activated neutrophils cause rheological changes that promote platelet adhesion and vessel activation. It is evident that the interplay between neutrophil, endothelial and platelet activation cannot be reduced to a single initiator of thrombosis, vessel stenosis or tissue fibrosis. Therefore, while blocking neutrophil-vessel interactions has shown therapeutic promise in reducing neutrophil-mediated vessel damage [81,88–92], pro-inflammatory neutrophil–platelet interactions are a compelling target for emerging therapeutics [93].

### 2.3. Platelets

Platelets are cell fragments of megakaryocytes that govern the haemostatic resolution of vascular wounds [94,95]*.* Thrombocytopenia and platelet depletion caused by genetic deficiencies or anticoagulants can provoke disorders such as haemophilia and interfere with the proper resolution of infection [96] and inflammation [97–99]. More recently, platelets have been recognized as drivers of inflammatory damage, working alongside circulating neutrophils to perpetuate inflammation [93,100,101]. During severe vessel damage, endothelial cell death [102] or vessel denudation [45] causes the exposure of underlying ECM. Circulating platelets readily bind to and activate on exposed collagen-rich ECM that lies beneath the endothelial layer to create a platelet plug.

While platelet plugs are vital for maintaining haemostasis within an injured vessel [103], in some cases, the haemostatic response can quickly become imbalanced by infiltrating immune cells, shifting the plug from haemostatic to thrombotic. In a haemostatic platelet plug, densely packed P-selectin positive platelets and a fibrin network form an ultra-dense thrombus core surrounded by more loosely packed P-selectin negative, minimally activated platelets at the luminal surface [103,104]. Once the healing cascade is compromised and thrombus formation becomes pathogenic, smaller vessels such as arterioles, capillaries and post-capillary venules are at a greater risk of occlusion and resultant tissue ischemia owing to thrombi taking up a larger relative proportion of the vessel [7,46,105]. Platelet thrombi formed after a traumatic or ischemia reperfusion injury can release the α-granule chemokine neutrophil-activating peptide 2 (NAP-2) to recruit circulating neutrophils to the injury site. These thrombi then act as migration points for circulating neutrophils [106], driving tissue fibrosis.

#### 2.3.1. Link to the inflammatory triangle

Adherent platelets have also been shown to guide neutrophils to inflammatory sites [54,107] or act as anchor points for secondary capture of circulating neutrophils [49,108–110]. This is especially apparent in arteries, where adherent platelets may be necessary for neutrophil recruitment [53], perhaps owing to high shear forces in the artery [7]. In addition to facilitating capture, activated platelets and platelet-derived soluble factors such as extracellular vesicles [111] can act as drivers of neutrophil and endothelial activation [83,102,110,112–114] and affect their migration behaviour [115]. Platelet–neutrophil–endothelial cell interactions are influenced by several factors, including the P-selectin-PSGL-1 axis [49,53,73,114,116–118] via phosphodiesterase type-4 [109] or Src family kinases [110,119], and the vWF-glycoprotein Ib*α* axis [48,84]. Similar to endothelial cells and neutrophils, shear stress seems to be a critical factor in achieving physiologically relevant platelet activation [120].

Upon activation, endothelial cells release Weibel-Palade bodies to the vascular surface. Weibel-Palade bodies are secretory granules that store platelet adhesion ligands P-selectin and vWF. Now available, P-selectin and vWF recruit additional circulating neutrophils and platelets [118], causing greater platelet and neutrophil recruitment and further aggravating the endothelium. In regions of endothelial denudation, this recruitment can be beneficial for wound healing [99]; however, when uncontrolled it can also lead to irreversible fibrotic damage.

### 2.4. Implications in tissue fibrosis

Paradoxically, immune cells recruited for repair can potentiate damage if the inflammatory stimulus is not resolved. Activated leucocytes, platelets and leucocyte-platelet complexes have been implicated as drivers of pulmonary and cardiovascular fibrotic diseases [121,122]. While not typically considered centre stage in fibrosis, dysregulated platelet and neutrophil accumulation play a critical role in aberrant tissue repair [123]. Platelet activation and degranulation are associated with myofibroblast proliferation and the overactive deposition of ECM components that results in tissue fibrosis [124]. Furthermore, activated platelets secrete large quantities of chemokines that are chemotactic for neutrophils and monocytes, triggering immune cells to migrate, activate and produce additional cytokines and enzymes that stimulate the production of transforming growth factor-β (TGF-β), a key mediator of myofibroblast formation, collagen deposition and fibrosis [125,126].

Neutrophil migration along activated endothelium is one of the first steps towards the resolution of acute inflammation; however, without proper clearance, neutrophil accumulation causes sustained, chronic inflammation. Extravasating neutrophils are activated by ECM proteins, leading to injury and remodelling of the surrounding tissue [127], as is seen in chronic obstructive pulmonary disease and atherosclerosis [128]. Excessive neutrophil activation, degranulation and respiratory burst activity damages surrounding tissues [129], in part by the release of matrix-degrading MMPs, toxic mediators such as ROS and reactive nitrogen species [2], and pro-inflammatory cytokines that activate and recruit other immune cells such as macrophages and T-lymphocytes. The reparative action by myofibroblasts in response to matrix degradation leads to the formation of a dense, disorganized fibrotic tissue [124].

## 3. Clinical manifestations

Several pro-fibrotic diseases have been linked to perturbations in the proposed inflammatory triangle. Select disease states are briefly discussed below.

### 3.1. Lung disease

The lungs are a particularly interesting platform for studying aberrant neutrophil–platelet interactions. Platelets have been shown to support recruitment and activation of neutrophils in the pulmonary capillaries in abdominal sepsis [124], acute lung injury [125], acute respiratory distress syndrome [125,130,131] and allergic inflammation [126]. Elevated platelet activation indices have been reported in idiopathic pulmonary fibrosis (IPF) patients [132], with anti-platelet drugs showing promise in alleviating IPF by reducing platelet activation and platelet-mediated neutrophil infiltration [133]. Recent insights into IPF have suggested that imbalanced endothelial activation plays a vital role in disease pathogenesis. Activated endothelium has been shown to secrete microparticles [134] and chemokines such as IL-8 into circulation, augmenting neutrophil recruitment and activation [135]. While the mechanisms underlying IPF are still unknown, vascular contributors such as these are an encouraging target.

### 3.2. Neointimal hyperplasia

Endothelial denudation, as occurs after balloon angioplasty and severe inflammatory damage [102], presents a unique environment for platelet–neutrophil interactions. Under these conditions, circulating platelets bind to and activate on exposed collagen [44,46,109,136,137] and release extracellular vesicles that have been shown to influence neutrophil activation states, causing the upregulation of platelet receptor CD41 [111]. Intimal hyperplasia, or fibrosis within the artery itself, has been clearly linked with increased inflammation [138]. This, in conjunction with studies relating platelet-mediated neutrophil binding and activation with thrombosis [49,54,102,108,109], suggests that more complex or combinatorial therapeutics are needed to reduce platelet- and neutrophil-mediated tissue damage.

### 3.3. COVID-19

Vascular inflammation and associated cytokine storm are known contributors to COVID-19 morbidities. These events are in part characterized by immune cell infiltration, NET formation [85] and dysregulated platelet activation [139], leading to hypercoagulopathy and vascular complications. Despite their role in hypercoagulopathy and the formation of pathogenic platelet–neutrophil complexes in this disease, platelets have paradoxically been shown to preserve endothelial integrity in severe COVID-19, with thrombocytopenia being associated with the impairment of platelet-dependent endothelium-protective mechanisms [140,141]. Therefore, as we learn more about this disease, it will be important to distinguish between restorative and noxious immune cell activation states when developing treatments.

Ventilator-induced damage likewise imbalances the inflammatory triangle. Severe COVID-19 patients that have developed acute respiratory distress syndrome require mechanical ventilation; while a necessary therapy, mechanical ventilation has been associated with exacerbated lung damage, referred to as ventilator-induced lung injury (VILI) [132,133]. Neutrophil infiltration and NET formation are elevated in patients with VILI [132], in part owing to platelet–endothelial interactions. Platelets have been reported to contribute to neutrophil recruitment in VILI by presenting leucocyte-binding proteins at the endothelial surface [142,143]. It is likely that VILI and inflammation-induced damage go hand-in-hand in these patients, with platelet–neutrophil interactions exacerbating cytokine storm-related morbidities.

## 4. Therapeutic advances

As initiators of the fibrotic cascade, neutrophils, platelets and activated endothelium have been investigated as potential therapeutic targets to combat vascular injury and occlusion and prevent the progression of fibrosis. For other vascular indications, established therapeutics such as rosuvastatin (statin; slows cholesterol production) [144] and ticagrelor (blood thinner; anti-platelet medication) [145] have exhibited a capacity to limit neutrophil binding, platelet aggregation and neutrophil–platelet aggregates and are discussed elsewhere [145–148]. Here, we focus on efforts to protect or regenerate the glycocalyx, and select mediators of endothelial, neutrophil and platelet-induced inflammatory damage.

### 4.1. Glycocalyx protection

While essential for healing processes, the inflammatory response can be devastating for damaged endothelium and underlying tissue if not kept under control. A pathological inflammatory response can lead to immune cell influx, altered vascular and tissue remodelling, and irreversible fibrotic damage, as is seen in ischemia reperfusion injury [149] and acute lung injury [150]. As a regulator of endothelial health, the inflammatory response and vascular permeability to macromolecules and immune cells, the endothelial glycocalyx presents a promising therapeutic target for a wide range of diseases without sacrificing the healing component of inflammation [151]. Here, we discuss recent efforts at preserving the glycocalyx that have shown promise in reducing vascular and underlying tissue damage. For a comprehensive review on glycocalyx preserving molecules, we refer the reader elsewhere [152,153].

Established drugs have been repurposed to combat glycocalyx damage and subsequent leucocyte and platelet adhesion, as is the case with the general anaesthetic sevoflurane. This drug is thought to function in part through upregulation of sialyltranferase, which in turn catalyses the transfer of sialic acid, an important mediator of oxidative stress [38,154]. Sevoflurane has shown promise in glycocalyx preservation in animal models of pulmonary ischemia reperfusion during lung transplant [155], cardiac ischemia reperfusion [156] and aortic damage by H_2_O_2_ [154], although limited efficacy has been shown in humans [157,158].

GAGs and proteoglycans such as heparin and heparan sulfate have shown promise as glycocalyx protecting therapeutics for many years [159]. Unfractionated and low molecular weight heparin are best known for their anticoagulant properties and use in treating or preventing thrombotic events by activating anti-thrombin III [160]. However, heparin has further been shown to possess anti-inflammatory properties, lending to improved vascular outcomes after inflammatory damage by limiting vascular permeability [161], neutrophil adherence and migration [63,162], and platelet adhesion [163], fibrin deposition [63], and thrombus formation [92,164,165]. As one of the oldest anticoagulants in clinical medicine, heparin and heparin-like products have been heavily studied [165–169]. More recently, low molecular weight heparin has been used to treat coagulopathy seen in severe COVID-19 patients [97,170,171], and nebulized unfractionated heparin is currently being evaluated in clinical trials for acute lung injury [171] and COVID-19 (ACCORD 2: A Multicentre, Seamless, Phase 2 Adaptive Randomisation Platform Study to Assess the Efficacy and Safety of Multiple Candidate Agents for the Treatment of COVID 19 in Hospitalised Patients, EudraCT number 2020-001736-95) [171]. While risks of thrombocytopenia and haemorrhagic shock limit the clinical use of this drug for inflammatory indications in its standard form, non-anticoagulant mechanisms of heparin are a promising pivot point for heparin-derived therapeutics [172].

Other promising molecules include sphingosine-1-phosphate and sulodexide. The signalling triggered by sphingolipid sphingosine-1-phosphate has been described as glycocalyx protective [173]. When paired with heparan sulfate, sphingosine-1-phosphate has been shown to regenerate the glycocalyx and restore inter-endothelial communication [21]. Sphingosine-1-phosphate analogues and receptor modulators have been tested clinically for a range of autoimmune and inflammatory diseases, including multiple sclerosis, psoriasis, acute stroke and inflammatory bowel disease [174,175]. However, prolonged exposure to sphingosine-1-phosphate has also been shown to enhance vascular leak and fibrosis after lung injury [176], therefore caution should be taken before employing sphingosine-1-phosphate modulating drugs to different organ systems and disease indications.

Sulodexide is a decades-old glycosaminoglycan mixture made up of 80% fast-moving heparin (iduronylglycosaminoglycan) and 20% dermatan sulfate that mitigates the bleeding risk of heparin-only therapeutics while maintaining anti-thrombotic potential [177,178]. Originally used for cardiac indications such as myocardial infarction [179], sulodexide has since been extended to other vascular disorders that manifest as endothelial dysfunction and glycocalyx damage, such as in type-2 diabetes mellitus patients [180] and in patients with venous ulcers [181]. Sulodexide has been shown to promote glycocalyx regeneration and improve animal survival after severe sepsis [22] and reduce the levels of collagen degrading MMP9 in patients with chronic vascular disease [24].

Taken together, the benefits seen with this class of molecules with respect to decreased neutrophil and platelet adhesion further suggest that restoring the function of the critical endothelial glycocalyx barrier can improve outcomes for fibrotic and thrombotic diseases that result from overexuberant interactions between endothelial cells, platelets and neutrophils.

### 4.2. Cytokine inhibitors

Recombinant human cytokines and cytokine receptors have been used as therapeutic targets to modulate inflammatory activity, driven in part by the pain patients experience as a result of acute and chronic inflammatory diseases [182,183]. Three core pro-inflammatory cytokines have been of particular interest in this realm: TNF-α, IL-1β, and IL-6. These cytokines act as key inducers of endothelial activation, neutrophil accumulation and activation, and platelet adhesion and degranulation, suggesting a potential benefit of inhibitors in regulating endothelial activation and the downstream consequences of endothelial dysfunction. There are currently several Food and Drug Administration (FDA) approved TNF-α, IL-1 and IL-6 inhibitors for clinical indications such as rheumatic diseases, Crohn's disease and cryopyrin-associated periodic syndromes (CAPS) [184].

TNF-α blockers have been successfully employed to combat fibrotic indications such rheumatoid arthritis and psoriasis [185,186]; unfortunately, these benefits are not ubiquitous for all fibrotic diseases, as was evidenced by their ineffectiveness against idiopathic pulmonary fibrosis [187]. Anti-TNF-α therapies for chronic heart failure have likewise been tested in clinical trials with little success [188], and potential beneficial effects on endothelial function in patients with inflammatory arthropathies are inconsistent [189].

IL-1β antagonists have shown greater potential in vascular inflammatory disorders, perhaps in part owing to the integral role of IL-1 in leucocyte and endothelial signalling. The FDA approved IL-1 agonists canakinumab (originally approved for CAPS), anakinra (originally approved for rheumatoid arthritis) and rilonacept (originally approved for CAPS) have shown promise in clinical trials of cardiovascular conditions such as pericarditis and recurrent ischemic events after myocardial infarction [190,191], but thus far none have been approved for these indications.

IL-6 is known to promote endothelial cell dysfunction and regulate leucocyte recruitment to the vascular wall [192]; therefore, it is unsurprising that FDA approved IL-6 blockers for arthritic conditions are of interest for treating off-label inflammatory conditions such as systemic sclerosis. Clinical trials of tocilizumab and sarilumab have recently begun as a treatment for ‘cytokine storm'-related morbidities in severe SARS-CoV-2 patients [70,193]. The anti-IL-6 receptor tocilizumab is particularly promising, as it has been shown to cause transient neutropenia without impairing host defence [194].

While cytokine inhibitors and cytokine receptor blockers may be beneficial in certain inflammatory conditions, the mixed results of this class of molecules suggest the need for combination therapies, or therapies more directed at the key cellular interactions.

### 4.3. Collagen protection

Severe endothelial damage can cause the exposure of the underlying collagen matrix, prompting rapid platelet adhesion. As described in this review, activated endothelium and collagen-adherent platelets provide an adhesive surface for neutrophil recruitment, eventually leading to tissue fibrosis; therefore, targeting exposed collagen could enhance anti-neutrophil–platelet therapies.

Several groups have designed collagen-targeting therapeutics to discourage platelet and/or neutrophil accumulation. Paderi and colleagues designed a proteoglycan mimetic consisting of collagen-binding peptides conjugated to a dermatan sulfate backbone that bound collagen, but not endothelium, of denuded arteries after balloon angioplasty [45]. Their therapeutic reduced *in vivo* platelet-induced vasospasm in the femoral artery as well as whole blood and platelet binding *in vitro*. Further, their studies showed that reduced platelet binding to the arterial wall correlated with reduced fibrosis or neointimal hyperplasia *in vivo*.

Similarly, McMasters *et al*. designed a thermoresponsive collagen-binding nanoparticle for effective systemic delivery of a mitogen-activated protein kinase-activated protein kinase 2 (MK2) anti-inflammatory peptide [43]. The authors show that nanoparticle delivery of the MK2 inhibitor *in vitro* reduced cellular binding to collagen surfaces, IL-6 levels in endothelial cells and smooth muscle cells, and platelet activation on a collagen matrix. Additional nanostructures have been developed to aid in drug delivery to exposed collagen. Collagen IV targeting nanoburrs [195] and nanofibres [196,197] have been designed to target angioplasty injured vasculature [195] and atherosclerotic plaques [197].

### 4.4. P- and E-selectin

Rather than targeting platelet deposition on denuded endothelium, Totani *et al*. have focused on preventing neutrophil deposition on adherent platelets [109], thereby reducing the likelihood of thrombosis within the vessel. Blockade of phosphodiesterase type-4 by rolipram, originally an anti-depressant drug, caused a reduction in neutrophil binding to fixed, activated platelets *in vitro* as well as adherent neutrophils along the denuded femoral artery *in vivo*. Adhesion was likewise lost on untreated P-selectin deficient platelets, further suggesting P-selectin as a mediator of platelet–neutrophil interactions.

P-selectin has been a focal point in anti-inflammatory therapeutics owing to the substantial role it plays in endothelial signalling, neutrophil and platelet recruitment to inflamed endothelium, and the formation of platelet-leucocyte aggregates. Competitive inhibitors of P- and E-selectin and their binding partners, PSGL-1, CD44 and E-selectin ligand 1, have been investigated as antagonists to neutrophil and platelet-mediated vascular damage. Monoclonal antibody therapy to P- and E-selectin have shown promise in combating platelet and neutrophil-mediated injury [198–200], as is seen with the FDA approved P-selectin antibody crizanlizumab, designed to address vaso-occlusive crises in sickle cell anaemia [201]. However, clinical trials of Inclacumab, the monoclonal antibody designed to bind P-selectin, evidenced that a major limitation of antibody therapy is the need for high doses for therapeutic effects [90,202], leading to high production costs [203,204].

Because of the role E- and P-selectin play in vaso-occlusive processes, small molecule inhibitors of these ligands are also under various stages of development. The pan-selectin antagonist Rivepansel (GMI-1070) was designed to combat vaso-occlusive crises in severe sickle cell anaemia by preventing the interaction of leucocytes and endothelium [205], but unfortunately failed to meet its phase 3 clinical trial endpoints. Clinical trials for GMI-1271, a small molecule inhibitor of E-selectin, is currently underway for treating venous thrombosis [206]. Exogenous recombinant human vimentin, a cytoskeletal structural protein and CD44-binding partner [207], has been shown to act as a competitive inhibitor of neutrophil binding to platelets and endothelial cells in a P-selectin-dependent manner [88]. Treatment with recombinant human vimentin reduced neutrophilia and acute lung injury scores in mice treated with sub-lethal doses of LPS. Clinical trials assessing the role of vimentin in sepsis, rheumatoid arthritis and renal transplant are currently underway. However, given that both increases and deficiencies of this protein can lead to vascular abnormalities [207,208], the effects of exogenous systemic administration will need to be extensively studied before vimentin is used as a therapeutic.

Selectins have been used as targeting modalities for glycan and polysaccharide-based molecules. Glycomimetics designed to bind selectins or selectin tetrasaccharide sialyl-Lewis^x^ are a new perspective on anti-inflammatory therapeutics [209], in part because of the anti-inflammatory nature of polysaccharides, glycosaminoglycans and proteoglycans. Recently, a selectin-targeting dermatan sulfate conjugate has been reported as reducing neutrophil and platelet interactions with inflamed endothelial cells *in vitro* and reduced thrombus formation *in vivo* [91,164]. Similarly, glycopeptide analogues of PSGL-1 effectively reduced neutrophil–platelet aggregates [80].

Polymer microcapsules coated with fucoidan, a complex polysaccharide that has been shown to slow blood clotting, have been proposed as a drug delivery tool targeted to inflamed vessels expressing P-selectin under high shear [210]. Polymer, glycosaminoglycan and polysaccharide-based therapeutics present indirect benefits in addition to competing for selectin binding. Bulky therapeutics could provide a steric boundary limiting neutrophil and platelet interactions with inflamed endothelium and exposed collagen. Dual targeting to inflamed endothelium and exposed collagen could likewise prevent the pathological accumulation of neutrophils, platelets and neutrophil–platelet aggregates in damaged vessels and thereby reduce immunothrombosis and tissue fibrosis.

As the initial points of capture for both neutrophils and platelets, selectin inhibition may provide a way to modulate thrombosis and fibrosis early on in disease progression. Therapeutics that blanket the adhesive endothelium, such as polymer-based molecules and glycoconjugates that are targeted towards upregulated selectins, may overcome limitations of monoclonal antibodies and recombinant proteins. Nonetheless, therapeutics will need to be studied on a case-by-case basis to maximize their efficacy as anti-inflammatory, anti-thrombotic or anti-fibrotic therapies.

## 5. Conclusion and perspective

**Figure 2.**
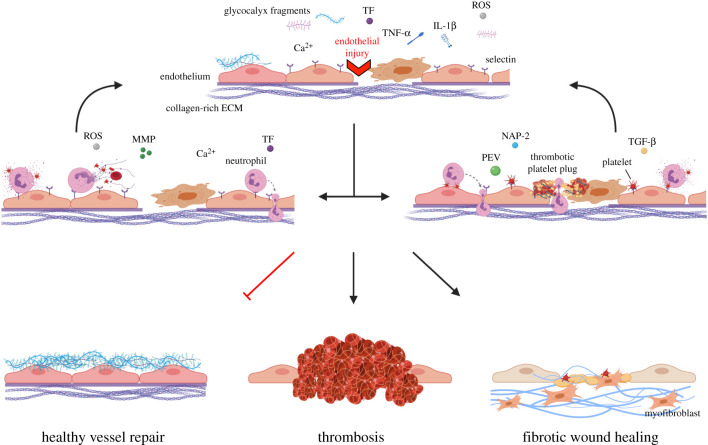
Neutrophil, platelet and endothelial contributions to vessel repair. In response to vessel injury, endothelial cells shed their protective glycosaminoglycan-rich layer, the endothelial glycocalyx, exposing upregulated selectins that have been mobilized to the cell surface. Activated endothelial cells secrete pro-inflammatory cytokines such as TNF-α and IL-1β that recruit circulating neutrophils and platelets to the damaged region. ROS production and dysregulated calcium homeostasis causes reductions in NO, enhanced cell contraction, vessel leakiness and the exposure of the underlying collagen-rich ECM. In some cases, neutrophils captured by upregulated selectins degranulate, releasing additional ROS and matrix-degrading MMPs. Adherent neutrophils act as secondary capture points for circulating platelets. Neutrophils undergoing NETosis capture red blood cells, platelets and fibrin, facilitating thrombosis development. Neutrophil activation results in further endothelial activation, which can lead to apoptosis of endothelial cells. In other cases, platelets anchor to upregulated vWF and P-selectin on the endothelial surface via GPIIbIIIa and PSGL-1. Adherent platelets secrete factors such as NAP-2, TGF-β and platelet extracellular vesicles (PEVs) that cause further endothelial activation, neutrophil recruitment, and promote the migration and proliferation of collagen-producing myofibroblasts. Platelets form a platelet plug at regions with severe endothelial damage, where denudation has occurred. Neutrophils infiltrate the platelet plug, causing an imbalance in the haemostatic response that shifts plug formation towards thrombotic. Activated platelets help guide crawling neutrophils through the thrombus or endothelium. Persistent or unresolved endothelial damage causes continued recruitment and activation of platelets and neutrophils, overwhelming the vessel and shifting the repair process towards thrombosis and fibrotic wound healing.

Neutrophils, platelets and activated endothelial cells each make substantial contributions to the initiation and perpetuation of vascular dysfunction. As such, many studies have been conducted to establish the relative contribution each of these makes to downstream thrombosis and fibrosis. When physiological redundancies are considered, it is likely that the relative contributions shift depending on the vascular environment in a coordinated effort to resolve an inflammatory stimulus (figure 2). Endothelial activation initiates the secretion of pro-inflammatory cytokines and shedding of glycocalyx components, prompting the recruitment of circulating immune cells. Upregulated endothelial E- and P-selectin capture circulating platelets and neutrophils. Adhesion of these inflammatory regulators propels further recruitment, eventually overwhelming the vessel's capacity for self-restoration and shifting the cellular environment towards thrombotic or fibrotic. Captured and rolling neutrophils capitalize on contracting, leaky endothelium, which presents extravasation points that facilitate neutrophil migration into the underlying tissue. Neutrophil activation augments endothelial activation through the release of degranulation products and, in severe cases, NET formation.

Severe endothelial activation exposes the underlying collagen-rich ECM, causing platelet recruitment beyond that triggered by selectin upregulation. Activated adherent platelets express platelet P-selectin, which can act as a congregation point for additional platelets or a secondary capture point for circulating neutrophils. Accumulation of platelets alone or platelet–neutrophil aggregates can result in vessel thrombosis and, in severe cases, vessel occlusion and tissue ischemia. In vessels more susceptible to fibrosis, platelet activation stimulates the production of pro-fibrotic cytokine TGF-β, pathological ECM secretion and downstream fibrosis.

Several therapeutic strategies have been implemented to combat the downstream effects of endothelial dysfunction. Each approach primarily targets one interaction to halt what appears to be a highly coupled group of interactions that, when unbalanced, results in pathological thrombosis and/or fibrosis. Conflicting outcomes suggest that no one therapeutic will be a panacea that resets the balance towards healthy regeneration and healing. Based on our current understanding of the coordination of endothelial cells, neutrophils, and platelets when the endothelium becomes dysfunctional, combination therapeutics or complex therapies that target multiple adhesion axes simultaneously may be most efficacious in treating thrombotic and fibrotic conditions.

Deeper understanding of these complex pathways is needed to develop therapeutic strategies for what is surely a large number of distinct pathological states that result from sterile inflammation, diffuse dysfunction that exists with metabolic syndrome, and the sudden and severe response to cytokine storm, like that seen as a result of severe COVID-19 infections, among others. Key discoveries such as the role of NO and ROS in endothelial vasoreactivity or, more recently, the role of platelets as drivers of thrombosis, inflammation and fibrosis, have been integral in strengthening our understanding of these complex phenomena. Furthermore, elucidating how interactions between neutrophils and endothelial cells can result in NET formation and eventual thrombus formation provides a new foundation from which to innovate and think about disease and healing. The community needs to remain open to the fact that the mechanisms involved in endothelial dysfunction, coupled with platelet and neutrophil interactions, are multipronged; therefore, combinatorial approaches to treatment may well be required. This mode of thinking will expand the potential of targeting not just intercellular signalling cascades, but also cell-cell and cell-ECM interactions to quickly alter the biological response to injury and disease.
